# Crop-Specific Responses to Cold Stress and Priming: Insights from Chlorophyll Fluorescence and Spectral Reflectance Analysis in Maize and Soybean

**DOI:** 10.3390/plants13091204

**Published:** 2024-04-25

**Authors:** Maja Mazur, Maja Matoša Kočar, Antun Jambrović, Aleksandra Sudarić, Mirna Volenik, Tomislav Duvnjak, Zvonimir Zdunić

**Affiliations:** 1 Agricultural Institute Osijek, Južno Predgrađe 17, 31000 Osijek, Croatia; maja.matosa@poljinos.hr (M.M.K.); antun.jambrovic@poljinos.hr (A.J.); aleksandra.sudaric@poljinos.hr (A.S.); mirna.volenik@poljinos.hr (M.V.); tomislav.duvnjak@poljinos.hr (T.D.); zvonimir.zdunic@poljinos.hr (Z.Z.); 2 Center of Excellence for Biodiversity and Molecular Plant Breeding, Faculty of Agriculture, University of Zagreb, Svetošimunska Cesta 25, 10000 Zagreb, Croatia

**Keywords:** C3 and C4 photosynthesis, chlorophyll *a* fluorescence, cold stress, crop-specific stress response, leaf spectral reflectance, priming effect

## Abstract

This study aimed to investigate the impact of cold stress and priming on photosynthesis in the early development of maize and soybean, crops with diverse photosynthetic pathways. The main objectives were to determine the effect of cold stress on chlorophyll *a* fluorescence parameters and spectral reflectance indices, to determine the effect of cold stress priming and possible stress memory and to determine the relationship between different parameters used in determining the stress response. Fourteen maize inbred lines and twelve soybean cultivars were subjected to control, cold stress, and priming followed by cold stress in a walk-in growth chamber. Measurements were conducted using a portable fluorometer and a handheld reflectance instrument. Cold stress induced an overall downregulation of PSII-related specific energy fluxes and efficiencies, the inactivation of RCs resulting in higher energy dissipation, and electron transport chain impairment in both crops. Spectral reflectance indices suggested cold stress resulted in pigment differences between crops. The effect of priming was more pronounced in maize than in soybean with mostly a cumulatively negative effect. However, priming stabilized the electron trapping efficiency and upregulated the electron transfer system in maize, indicating an adaptive response. Overall, this comprehensive analysis provides insights into the complex physiological responses of maize and soybean to cold stress, emphasizing the need for further genotype-specific cold stress response and priming effect research.

## 1. Introduction

Cold stress poses a significant threat to plant growth, particularly in the early stages of development when temperatures fall below the range optimal for their growth and physiological functions. This environmental challenge not only affects physiological processes but also has morphological consequences for crops. The stress condition leads to reduced seed emergence, impaired seedling establishment, leaf wilting, and chlorosis [1]. In severe cases, it can even result in seed rotting, leaf necrosis, and plant death [1]. This environmental challenge disrupts various metabolic processes within the plant, affecting photosynthesis, nutrient uptake, and overall metabolic functions [2,3,4]. Plants, facing the unpredictable and temporary nature of cold stress, have evolved resource-efficient stress response mechanisms that activate only in the presence of stress [5]. Moreover, certain plants exhibit improved performance in subsequent or repeated abiotic stress (severe heat, cold, drought, or osmotic stress) due to their previous exposure to the same stress, known as stress priming [5,6,7,8,9,10]. This phenomenon has been observed in various plants displaying responses to drought memory [11,12,13,14,15,16] and salt stress memory [17]. Plants exhibiting a memory response demonstrate distinct physiological changes compared to non-primed plants, including decreased stomatal conductance, reduced photosynthesis, enhanced relative water content, elevated chlorophyll content, increased maximum quantum efficiencies of photosystem II, and better performance against oxidative damage, lower H_2_O_2_, and increased ABA contents, as evidenced by studies by Sintaha et al. [16], Ding et al. [11], Wang et al. [12], and Li et al. [18].

Low temperatures significantly impact photosynthesis, with variations in overall photosynthetic capacity among species and cultivars [19,20]. Based on anatomical differences, plants are categorized as either C3 or C4, exemplified by maize (*Zea mays* L.) (C4) and soybean (*Glycine max* L. Merr.) (C3), influencing their growth patterns, yield potential, and responses to different climates [19]. Maize’s C4 pathway efficiently reduces photorespiration and water loss, making it suitable for hot and dry conditions, while soybean’s C3 pathway is less effective in minimizing water loss and photorespiration, making it adaptable to temperate climates [19]. Both crops are highly sensitive to low-temperature conditions, particularly during early growth stages, with cold stress causing the impairment of growth, development, and yield occurring when temperatures fall below 15 °C for soybean [21] and below 12 °C for maize [22]. Understanding these anatomical and physiological differences is crucial for comprehending plants’ adaptation strategies, especially in agriculture. Scientists actively research the mechanisms of priming, aiming to create crop varieties with improved tolerance to cold stress, contributing to food security and sustainable agriculture in regions prone to temperature fluctuations. Exposing plants to low non-freezing temperatures can increase freezing tolerance. This is known as cold acclimation [23]. However, maize and soybean are unable to acclimatize to cold stress when ice forms in their tissue [24]. Experimental studies have shown that plant tolerance to chilling temperatures can be enhanced not only through acclimation but also by cold priming through the experience of individual short stress events. While it does not alter cold sensitivity itself, cold priming positively modifies the response to cold [25]. Initially observed in seeds [26,27,28,29,30], cold priming has been increasingly documented in vegetative tissues in recent years [25]. The impact of exposing plants to cold stress and the ability of plants to memorize stress and enhance their response to repeated stress have been examined in *Arabidopsis* [31] and wheat [32,33]. In contrast to the well-established field of seed priming, the analysis of cold priming in vegetative stages is a relatively new and emerging topic, gaining growing attention.

Physiological changes associated with reduced photosynthesis can be detected through chlorophyll fluorescence measurements, even before visible symptoms emerge [34]. In recent years, the integration of hyperspectral techniques has become increasingly essential in precision agriculture for rapid assessments of crop physiological characteristics [35]. These advanced techniques enable a more comprehensive analysis of the plant’s response to cold stress by capturing a wide range of spectral information. A plant’s reflectance spectra’s variations provide valuable insights into leaf structure, pigment content, and elemental composition alterations under different biological or abiotic factors [36]. These spectral variations can serve as sensitive indicators of plant health, making them a powerful tool for monitoring and diagnosing cold stress in plants before visible signs become apparent. Chlorophyll *a* fluorescence and spectral reflectance measurements hold additional significance as both methods are non-destructive, which represents an exceptionally crucial aspect in agriculture. The non-destructive nature of these methods allows for monitoring physiological changes in plants without the need for plant destruction or sampling.

Numerous studies have investigated the impact of cold stress on chlorophyll *a* fluorescence and leaf reflectance across various crop species [37,38,39,40,41,42,43,44]. For instance, research on winter oilseed rape cultivars under cold stress has shown declines in the maximal fluorescence and electron transport rate, coupled with changes in quantum yield and non-photochemical quenching [37]. Similarly, chickpea genotypes experienced a reduced maximum quantum efficiency and operating efficiency of photosystem II (PSII) under freezing stress [38]. Cold-tolerant crops such as wheat and rye demonstrate strong photosynthetic recovery at low temperatures after acclimation to cold conditions [39,40]. Soybean exhibited a decreased maximum quantum efficiency of PSII in the dark-adapted state and impaired photosynthesis due to cold stress [41,42]. Variations in absorbance, reflectance, and transmittance under cold stress in rice have been used to distinguish between tolerant and sensitive genotypes, with the range of 525–535 nm proving the most stable and wavelengths above 700 nm being the most sensitive in the reflectance curve [43]. In maize, cold stress mainly affected reflectance between 500 and 600 nm, as well as around 700 nm, with spectral indices indicating decreased chlorophyll levels and an increased carotenoid/chlorophyll ratio in cold-exposed plants [44].

The main objectives of this study were as follows: (i) to determine the effect of cold stress on chlorophyll *a* fluorescence parameters and spectral reflectance indices in the early development of maize and soybean, crops with diverse photosynthetic pathways; (ii) to determine the effect of cold stress priming and possible stress memory in maize and soybean; and (iii) to determine the correlation between changes in chlorophyll *a* fluorescence parameters, spectral reflectance indices, and biomass accumulation affected by cold stress. Understanding the impact of low temperatures, plant response, the priming effect, and plant stress memory facilitates the selection of cold stress-tolerant genotypes. Cold-stress maize and soybean genotypes enable early sowing, thus providing a possibility for avoiding combined summer stresses of high temperatures and drought in the most sensitive development stages.

## 2. Results

### 2.1. The Effect of Cold Stress on the Rapid Chlorophyll a Fluorescence Induction Kinetic Curve (OJIP)

**Figure 1:**
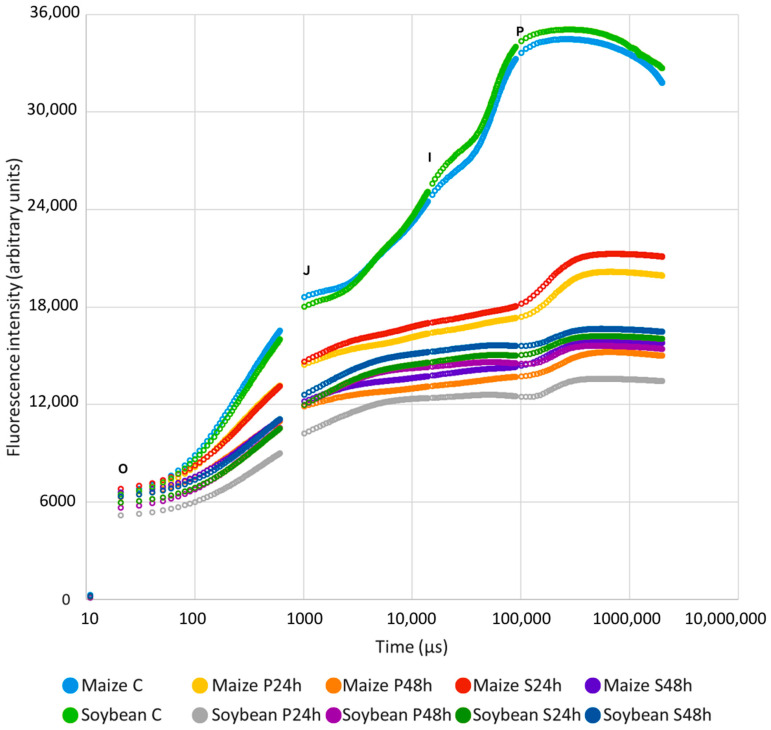
Effect of cold stress on OJIP fluorescence induction curve. Transient curves represent means of all genotypes (eight measurements per genotype, i.e., n = 112 for maize; n = 96 for soybean).

Cold stress significantly affected the OJIP fluorescence transient curves of both maize and soybean (Figure 1). In maize, the curve decreased with the prolonged duration of cold stress in both cold stress treatments. Conversely, in soybean, an increase in the curve was observed after 48 h of cold stress compared to 24 h of stress, evident in both cold treatments and more pronounced in P treatment. Furthermore, a decrease in fluorescence intensity is observable already at the O step in soybean, while in maize, the change was less pronounced at the O step compared to other steps of the OJIP curve (Figure 1).

### 2.2. Crop Type and Cold Stress Duration-Dependent Changes in Chlorophyll a Fluorescence Parameters

**Figure 2:**
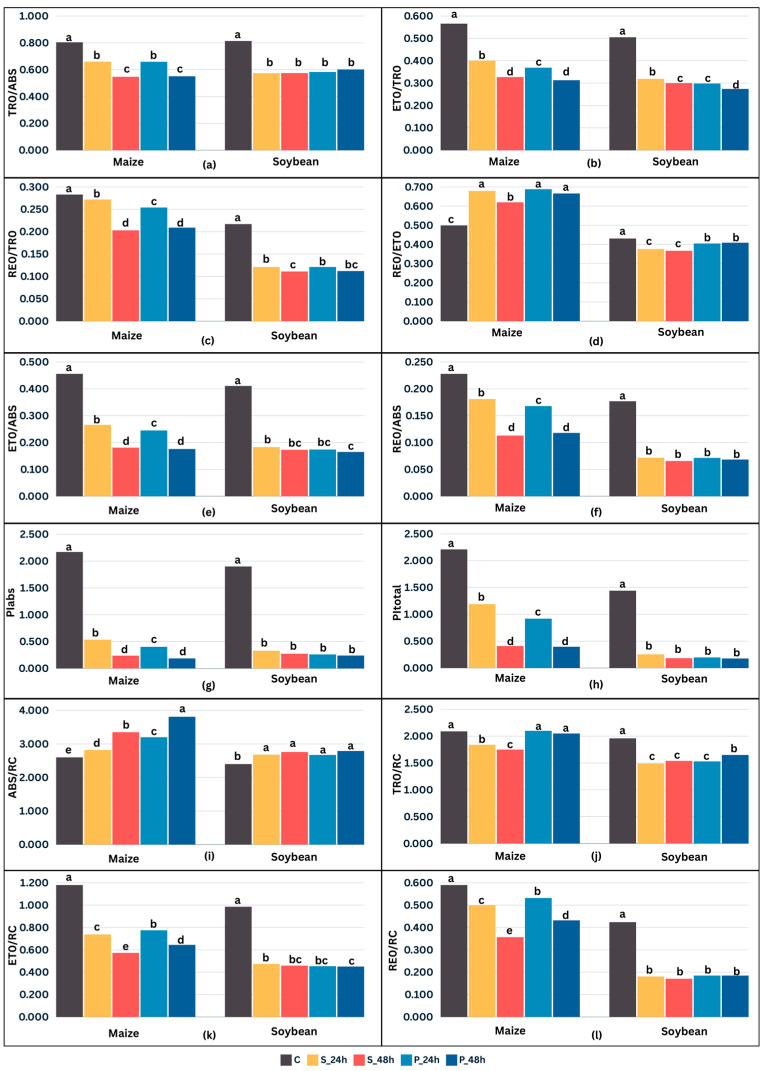
Chlorophyll *a* fluorescence parameters (maximum quantum yield of primary PSII photochemistry, TR_0_/ABS (**a**); efficiency with which PSII trapped electron is transferred from Q_A_^−^ to PQ, ET_0_/TR_0_ (**b**); efficiency with which PSII trapped electron is transferred to final PSI acceptors, RE_0_/TR_0_ (**c**); efficiency with which electron from PQH_2_ is transferred to final PSI acceptors, RE_0_/ET_0_ (**d**); quantum yield of electron transport from Q_A_^−^ to PQ, ET_0_/ABS (**e**); quantum yield of electron transport from Q_A_^−^ to final PSI acceptors, RE_0_/ABS (**f**); performance index on absorption basis, PI_abs_ (**g**); total performance index on absorption basis, PI_total_ (**h**); apparent antenna size of active PSII, ABS/RC (**i**); maximum trapped exciton flux per active PSII, TR_0_/RC (**j**); flux of electrons transferred from Q_A_^−^ to PQ per active PSII, ET_0_/RC (**k**); and flux of electrons transferred from Q_A_^−^ to final PSI acceptors per active PSII, RE_0_/RC (**l**)) measured on 14 dent maize inbred lines (FAO 500–600) and 12 soybean cultivars (0–I maturity group) in control (C), 24 h cold stress without priming (S_24 h), 48 h cold stress without priming (S_48 h), 24 h cold stress after two-days priming (P_24 h), and 48 h cold stress after two-days priming (P_48 h). Values presented are means of all genotypes (eight measurements per genotype, i.e., n = 112 for maize; n = 96 for soybean). Different letters above bars indicate significant difference at *p* < 0.05 according to Tukey’s HSD test for maize and soybean separately.

An analysis of variance revealed that treatment was a significant (*p* < 0.05) source of variation for all chlorophyll *a* fluorescence parameters, while maize and soybean significantly (*p* < 0.05) differed in all fluorescence parameters except the maximum quantum yield of primary PSII photochemistry (TR_0_/ABS). Among all chlorophyll *a* fluorescence parameters, only photon absorption (ABS/RC) values were higher as a result of cold stress in both crops (Figure 2i). The efficiency with which an electron is transferred from PQH_2_ to the final PSI acceptors (RE**_0_**/ET_0_) was higher in cold stress compared to C but only in maize (Figure 2d). In both maize and soybean, there were no significant differences between TR_0_/ABS in S and P. The values decreased with the duration of cold stress for maize but not for soybean (Figure 2a). In general, the difference between S and P was more evident in maize compared to soybean. For the efficiency with which a PSII trapped electron is transferred from Q_A_^−^ to PQ (ET_0_/TR_0_), the efficiency with which a PSII trapped electron is transferred to final PSI acceptors (RE_0_/TR_0_), the quantum yield of electron transport from Q_A_^−^ to PQ (ET_0_/ABS), the quantum yield of electron transport from Q_A_^−^ to final PSI acceptors (RE_0_/ABS), and performance indices (PI_abs_, PI_total_) in maize, the values were significantly (*p* < 0.05) lower in P compared to S and C initially, i.e., after the first 24 h of cold stress. However, after 48 h, they were at the same level as in S after 48 h (Figure 2b,c,e–h). On the other hand, ABS/RC had higher values in P compared to S in maize, and they continued to increase with stress duration (Figure 2i). The RE_0_/ET_0_ measured after 48 h of cold stress had significantly (*p* < 0.05) higher values in P compared to S in maize (Figure 2d). The same was true for the electron trapping efficiency (TR_0_/RC), the flux of electrons transferred from Q_A_^−^ to PQ per active PSII (ET_0_/RC), and the flux of electrons transferred from Q_A_^−^ to final PSI acceptors per active PSII (RE_0_/RC), regardless of the stress duration (Figure 2j–l). In soybean, there were no differences between P and S for most chlorophyll *a* fluorescence parameters, except ET_0_/TR_0_ with lower values in P than in S (Figure 2b), RE_0_/ET_0_ with higher values in P than in S regardless of the cold stress duration (Figure 2d), and TR_0_/RC with higher values in P than in S but only after 48 h of cold stress (Figure 2j).

### 2.3. Crop Type and Cold Stress Duration-Dependent Changes in Spectral Reflectance Indices

**Figure 3:**
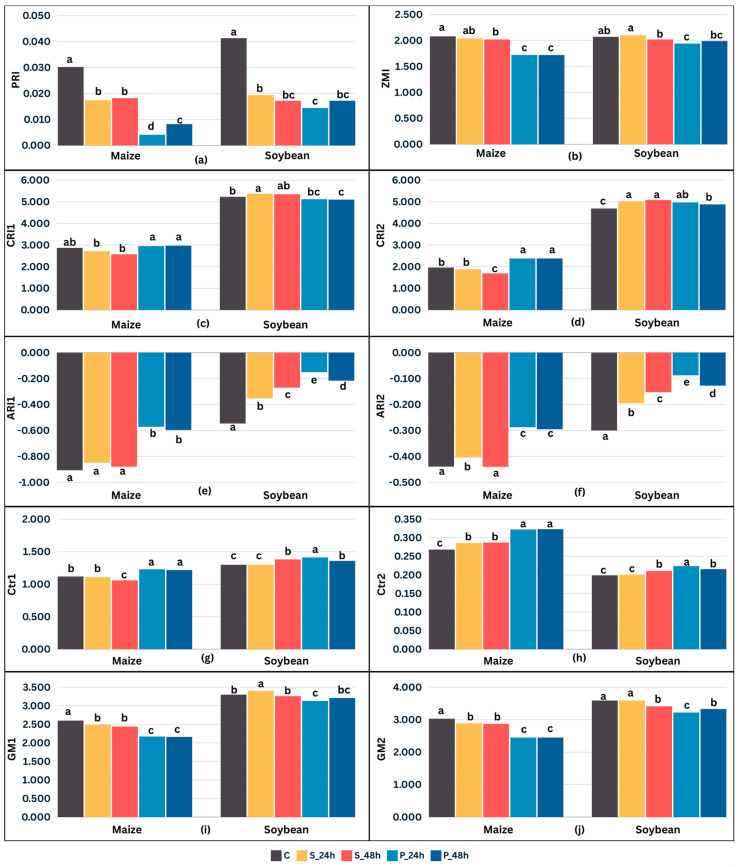
Chosen leaf spectral reflectance indices (photochemical reflectance index, PRI (**a**); Zarco-Tejada and Miller index, ZMI (**b**); carotenoid reflectance index, CRI1 and CRI2 (**c**,**d**); anthocyanin reflectance index, ARI1 and ARI2 (**e**,**f**); Carter index, Ctr1 and Ctr2 (**g**,**h**); and Gitelson and Merzlyak index, GM1 and GM2 (**i**,**j**)) for 14 dent maize inbred lines (FAO 500–600) and 12 soybean cultivars (0–I maturity group) in control (C), 24 h cold stress without priming (S_24 h), 48 h cold stress without priming (S_48 h), 24 h cold stress after two-days priming (P_24 h), and 48 h cold stress after two-days priming (P_48 h). Values presented are means of all genotypes (eight measurements per genotype, i.e., n = 112 for maize; n = 96 for soybean). Different letters above bars indicate significant difference at *p* < 0.05 according to Tukey’s HSD test for maize and soybean separately.

An analysis of variance revealed significant (*p* < 0.001) differences between treatments and crop species for all examined reflectance indices (Figure 3). The photochemical reflectance index (PRI) emerged as an index with the most substantial change in stress treatments for both crops, displaying the greatest reduction in values compared to control conditions (41.1% and 79.5% for maize, 55.7% and 61.7% for soybean in S and P, respectively), with P being significantly different compared to S only in maize (Figure 3a). Although PRI is sensitive to variations in carotenoid pigments, carotenoid reflectance indices (CRI1 and CRI2) did not exhibit changes as drastic as the PRI (Figure 3c,d). CRI1 and CRI2 showed very small changes in stress treatments for soybean. For maize, a slight increase was observed in P compared to C. However, there was a slight decrease in CRI2 in S after 48 h compared to C (Figure 3d). Maize and soybean significantly (*p* < 0.001) differed in the estimated values of carotenoid and anthocyanin content under control conditions. Indices related to the anthocyanin content in leaves, anthocyanin reflectance indices (ARI1 and ARI2), significantly (*p* < 0.05) increased compared to the control in both stress treatments for soybean (43.0% and 66.5% for ARI1, and 42.3% and 64.3% for ARI2 in S and P, respectively) and only in P for maize (35.3% ARI1, 33.5% ARI2; Figure 3e,f). In contrast, a slight decrease was observed for the Zarco-Tejada and Miller index (ZMI) related to the chlorophyll content in the leaf (Figure 3b).

The Carter index Ctr1 was higher compared to C in S after 48 h of cold and P for both stress durations in soybean. Similarly, in maize, Ctr1 was higher in P but lower in S (Figure 3g). The Carter index Ctr2 was higher in all stress treatments compared to C for both crops, except for S after 24 h of stress in soybean (Figure 3h). Although 48 h of S caused a significant (*p* < 0.001) decrease in Ctr1 compared to C for maize, there were no differences between S and P after 24 h. On the other hand, both stress durations caused a significant (*p* < 0.001) Ctr1 increase in P compared to C (Figure 3g). On the other hand, all stress treatments, regardless of the duration, were significantly (*p* < 0.001) higher compared to C for maize Ctr2 (Figure 3h). For soybean, both Ctr1 and Ctr2 had similar trends: the values in P increased significantly (*p* < 0.001) compared to C regardless of the duration, and both indices remained stable after 24 h of S, but they increased significantly (*p* < 0.001) compared to C after 48 h of S (Figure 3g,h). Gitelson and Merzlyak indices (GM1 and GM2) showed small but significant (*p* < 0.05) decreases in cold stress treatments compared to the control, with the exception of P for maize (16.9% and 19.1% decrease, respectively). Furthermore, no significant difference was determined in soybean GMI1 between C and stress treatments after 48 h or in GMI2 between C and S after 24 h (Figure 3i,j).

### 2.4. Effects of Cold Stress on Biomass Accumulation

**Figure 4:**
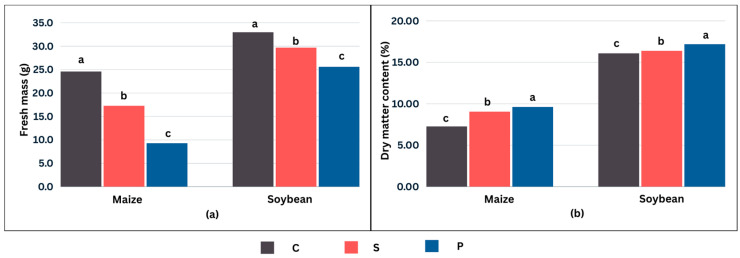
Fresh mass (**a**) and dry matter content (**b**) for 14 dent maize inbred lines (FAO 500–600) and 12 soybean cultivars (0–I maturity group) in control (C), cold stress without priming (S), and cold stress after two-days priming (P). Values presented are means of all genotypes. Letters above bars show no meaningful difference at *p* < 0.05 according to Tukey’s HSD test for maize and soybean separately.

The aboveground biomass and dry matter content under control conditions and different cold stress treatments are shown in Figure 4. The fresh mass of both crops exhibited a clearly decreasing trend with increasing low temperature duration. The aboveground biomass of maize was reduced by 29.7% in S and 62.2% in P. In soybean, the reduction in fresh mass was 10.0% and 22.4%, respectively. The cumulative effect of the duration of low temperatures in P is also evident in the dry matter content. Both crops increased the dry matter content under cold stress.

### 2.5. Changes in Spectral Reflectance Signature under Cold Stress

**Figure 5:**
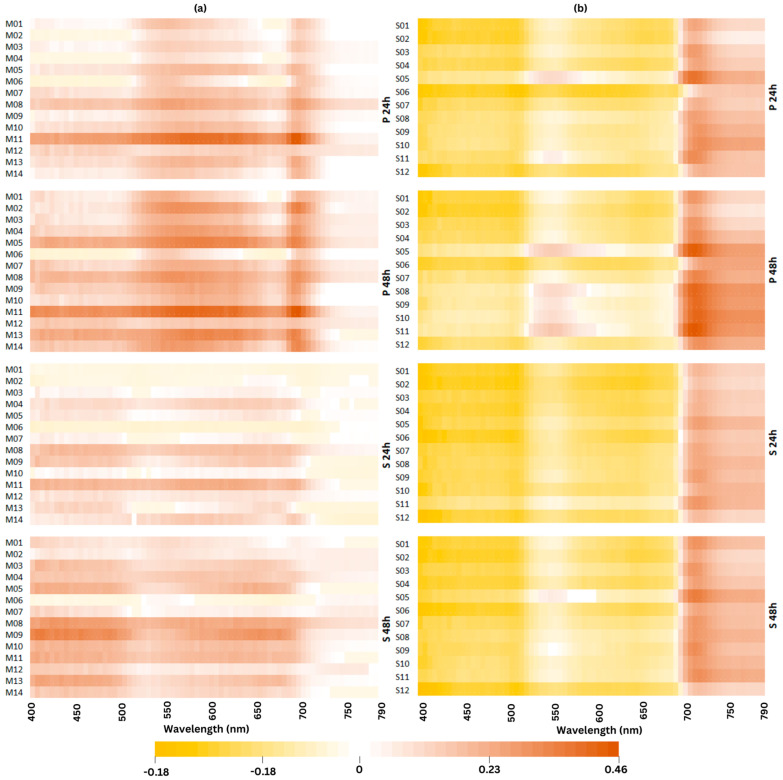
A heatmap of changes in the spectral reflectance across examined maize (**a**) and soybean (**b**) genotypes calculated as the percentage change in the wavelength level under stress compared to the control. The color intensity indicates the magnitude of the change compared to the control.

An analysis of variance of single wavelength reflectance (400–790 nm) revealed numerous wavebands exhibiting significant (*p* < 0.001) genotype and treatment effects in both maize and soybean. The spectral reflectance patterns of plants subjected to P and S treatments displayed variations across genotypes and over time (Figure 5). The effect of P treatment on the spectral pattern was more pronounced in maize (Figure 5a) than in soybean (Figure 5b). Noteworthy shifts in spectral reflectance for most maize genotypes were identified around wavelengths of 550 nm and 690 nm in P, while reflectance disparities in S were more genotype-specific (Figure 5a). The average reflectance signatures of maize predominantly increased compared to their initial state in C, whereas in soybean, the reflectance decreased relative to C at wavelengths below 680 nm (Figure 5a,b).

### 2.6. Correlation Analysis

**Figure 6:**
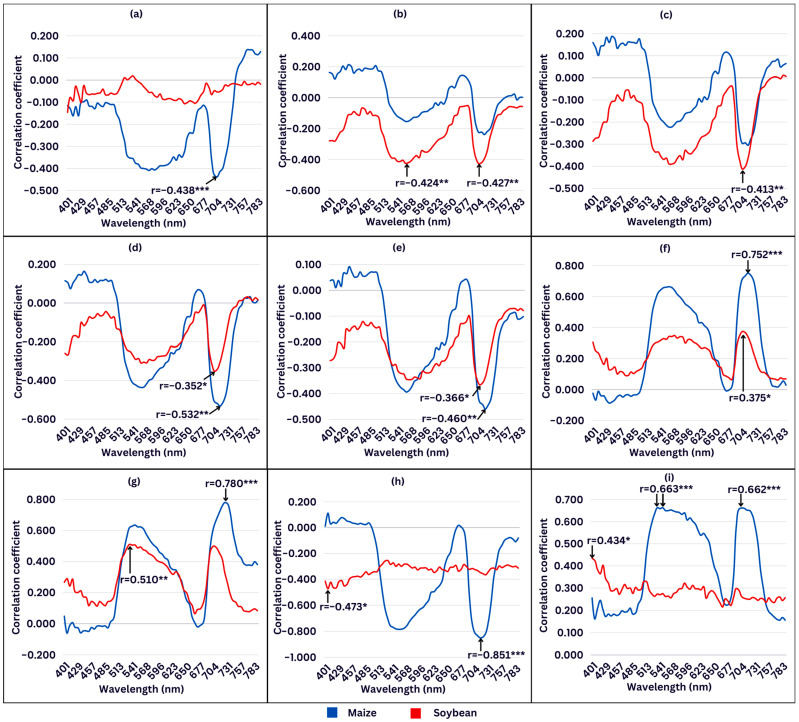
Pearson’s correlation analysis between changes in reflectance measured at different wavelengths during cold stress in maize and (**a**) maximum quantum yield of primary PSII photochemistry, TR_0_/ABS; (**b**) efficiency with which PSII trapped electron is transferred to final PSI acceptors, RE_0_/TR_0_; (**c**) quantum yield of electron transport from Q_A_^−^ to final PSI acceptors, RE_0_/ABS; (**d**) performance index on absorption basis, PI_abs_; (**e**) total performance index on absorption basis, PI_total_; (**f**) apparent antenna size of active PSII; (**g**) maximum trapped exciton flux per active PSII, ABS/RC; (**h**) fresh mass, FM; and (**i**) dry matter content, DMC. *, **, and *** represent correlations with *p* < 0.05, *p* < 0.01, and *p* < 0.001, respectively.

Changes in chlorophyll *a* fluorescence parameters during cold stress in maize showed weak-to-strong correlations with changes in relative reflectance measured at specific wavelengths (Figure 6a–g). For soybean, weak negative and weak-to-moderate positive correlations were observed (Figure 6a–g). Although statistically significant correlations were noted at approximately similar wavelength ranges for both crops, it is interesting to note that not all the same fluorescence parameters were significantly correlated with reflectance measurements. TR_0_/ABS showed significant correlations with changes in reflectance in maize, whereas there was no significant correlation of this parameter in soybean (Figure 6a). Conversely, RE_0_/TR_0_ and RE_0_/ABS showed significant correlations with changes in reflectance in soybean and no significant correlation in maize (Figure 6b,c). Moreover, it can be seen that most chlorophyll *a* fluorescence parameters exhibit their maximum correlation coefficient at the same wavelength range in both crops, with maize tending to slightly shift to longer wavelengths. The highest correlation coefficients were found in the spectral range between 520 and 600 nm and 690 and 730 nm, with the strongest one (r = 0.780) at 725 nm (Figure 6g).

Biomass traits showed a moderate-to-high correlation with changes in relative reflectance measured at specific wavelengths under cold stress in maize (Figure 6h,i). On the contrary, low correlations were found between biomass traits and changes in spectral reflectance in soybean. Fresh mass was negatively correlated with changes in reflectance, while dry matter content was positively correlated (Figure 6h,i). Moreover, each biomass trait exhibited its maximum correlation coefficient at a different wavelength in maize and soybean. The highest significant correlation coefficients for fresh mass were found at 715 nm (r = −0.851) for maize and at 406 nm (r = −0.473) for soybean (Figure 6h). Similarly, the correlation between changes in dry matter content and reflectance showed two significant regions in maize at 530–600 nm and 700–725 nm, while in soybean, significant correlations were found at 400–410 nm.

## 3. Discussion

Based on the chlorophyll *a* fluorescence parameters and leaf spectral indices, we investigated and compared the changes in the fluorescence characteristics of PSII and spectral properties of maize, as C4, and soybean, as a C3 plant, under cold stress. The analysis of OJIP kinetics in the present study showed significant differences in leaf photochemistry between the tested plant species (Figure 1). Chilling significantly affected all studied photosynthetic parameters, but stress duration had a significant effect only on maize (Figure 2). A large PI_abs_ and PI_total_ reduction, noted for both tested crops (Figure 2g,h), signalized an overall downregulation of PSII-related specific energy fluxes and efficiencies as a physiological response to stress [45,46,47,48,49]. The decrease in TR_0_/ABS (Figure 2a) and an increase in ABS/RC (Figure 2i) as a result of cold stress suggested an inactivation of the reaction centers (RCs), i.e., a transformation of active RCs to silent RCs, favoring higher energy dissipation as heat and fluorescence [49,50]. An acute TR_0_/ABS reduction, noted in the presented research (Figure 2a), is known to indicate PSII photoinhibition under cold stress [34,42,51,52]. Furthermore, it has been reported that chilling causes a reduction in TR_0_/ABS and can discriminate cold-tolerant from cold-sensitive genotypes [53,54]. ET_0_/TR_0_, RE_0_/TR_0_, ET_0_/ABS, and RE_0_/ABS (Figure 2a–c,e,f) decreased the same as TR_0_/ABS (Figure 2a) in both tested crops, indicating a further impairment of the electron transport chain, i.e., lower efficiency with which a PSII trapped electron is transferred to final PSI acceptors [55,56]. Nevertheless, RE_0_/ET_0_, which can indicate an upregulation or modification of the electron transport chain, increased in cold-stressed maize (Figure 2d). Although slightly lower compared to the control in cold-stressed soybean, it was positively affected by priming, stabilizing it almost to the level of the control after 48 h of cold stress. The increase in RE_0_/ET_0_ can be an adaptive response protecting the photosystems from further stress-induced damage. Overall, the difference in chlorophyll *a* fluorescence parameters between cold stress with and without priming was more pronounced for maize than soybean but mostly after the first 24 h of cold stress (Figure 2). Although cold stress generally initiated the inactivation of RCs, evident by the increase in ABS/RC [57,58], ABS/RC was significantly higher in primed maize compared to the control and stress without priming (Figure 2i), indicating a cumulative negative effect of cold stress length. As the RCs were inactivating and the amount of energy trapping per absorption was decreasing, the part of the absorbed excitation energy “captured” by the chlorophyll molecules of the reaction centers (TR_0_/RC) was reduced as well (Figure 2j). TR_0_/RC denotes the energy that reduces the electron acceptor Q_A_ to Q_A_^−^ and then reoxidizes it again to Q_A_, which forms the basis of a photosynthetic electron transfer system [59]. Although more RCs were inactivated in primed maize compared to the control and stress without priming, the electron trapping efficiency stayed stable, i.e., the electron transfer system was not impaired, as indicated by TR_0_/RC being at the same level as in the control. The other two specific energy fluxes, ET_0_/RC (Figure 2k), denoting the flux of electrons transferred from Q_A_^−^ to PQ per active PSII, and RE_0_/RC (Figure 2l), denoting the flux of electrons transferred from Q_A_^−^ to final PSI acceptors per active PSII, decreased, which was expected as their derivation is grounded in TR_0_/RC [55,56]. Again, primed maize had higher values of the mentioned parameters compared to stress without priming, indicating that electron transfer was less impaired.

The visible reflectance properties of maize and soybean leaves differed in control conditions, which can be attributed to differences in the cellular structure in monocots and dicots [60]. Chilling temperatures significantly influenced leaf reflectance (Figure 3 and Figure 5), with the VIS range primarily affected by plant pigments such as chlorophyll, anthocyanin, and carotenoids, which react to visible light [61]. Reflectance in the NIR region decreases due to the altered cell structure, while reflectance in the red region increases leading to a decrease in chlorophyll-related indices under low temperature [62]. The reflectance indices estimating chlorophyll content were significantly higher in plants continuously grown in control conditions relative to those exposed to cold (Figure 3). The ZMI responded to cumulative stress duration (Figure 3b). The ZMI is generally positively correlated with the total concentration of leaf chlorophyll [63,64], indicating that a decreased ZMI suggests a lower chlorophyll content in cold-stressed plants. Reduced chlorophyll levels lead to a lower maximum quantum efficiency of photosystem II photochemistry [65]. Chilling-induced photosynthesis reduction results in increased xanthophyll de-epoxidation, known for protecting plants from stress by absorbing the excess light not being used by the photosynthetic apparatus. The PRI was lower in plants subjected to cold stress (Figure 3a). A reduction in the PRI associated with cold stress was evidenced in maize hybrids and inbred lines, suggesting an increased carotenoid/chlorophyll ratio and/or xanthophyll pigment de-epoxidation [44]. The PRI assesses the reflectance of green vegetation and therefore is sensitive to variations in carotenoid pigments, particularly xanthophyll, which is closely related to photosynthetic efficiency and serves as an essential mechanism for rapid photosynthetic adaptation to stressors [66]. Variations in carotenoid pigments serve as indicators of the energy assimilated during photosynthesis, representing either the efficiency with which light is utilized or the rate at which green vegetation absorbs carbon dioxide. This is manifested through leaf fluorescence and the process of photosynthesis [67]. Therefore, the PRI has been utilized to detect various stresses in crops [68,69].

Cold stress also affected the estimation of carotenoids and anthocyanin content (Figure 3c–f). Increased carotenoids relative to chlorophyll can enhance photoprotection by dissipating excess excitation energy that cannot be used in photosynthesis, having a critical role as photoprotective compounds limiting membrane damage during abiotic stresses [70]. Known to increase in stressful conditions, carotenoids are bound in the pigment–protein complexes of the photosynthetic apparatus according to a conserved stoichiometry that is not only photosynthetic pathway-specific but often also species-specific [71,72,73].

However, estimating the carotenoid content from reflectance is challenging due to absorption peak overlap between chlorophyll and carotenoids and higher chlorophyll concentration [36]. Anthocyanin has antioxidant properties, and its production is often associated with stress responses in plants as it is known to scavenge the stress-induced reactive oxygen species (ROS), thus aiding in plant protection [74,75,76]. They protect plants from the adverse impacts of excessive light by absorbing high-energy quanta [77] and stimulating the plant’s antioxidant mechanisms, thereby counteracting reactive oxygen species and radicals [36]. ARI1 and ARI2 were suggested by Gitelson et al. [78] as non-invasive techniques for predicting anthocyanin levels. ARI1 and ARI2 increased with prolonged cold stress, with higher values observed in plants exposed to stress after priming compared to stress without priming (Figure 3e,f). Interestingly, after 48 h of cold stress post-priming, both indices slightly decreased in both crops compared to the 24 h duration of P. Anthocyanin estimation faces similar challenges as carotenoids due to absorption overlap with chlorophyll. As C4 plants exhibit different stress responses compared to C3 plants, the difference in their anthocyanin levels was expectedly noted in some recent studies [75,79].

Low temperatures reduced the growth and development of both crops (Figure 4). The impact of cold stress on aboveground biomass was more pronounced in maize. Priming had a detrimental effect on biomass accumulation. The diminished growth of maize can be attributed to the vulnerability of its C4 photosynthetic mechanism to low temperatures. This susceptibility can, in turn, be influenced by the upregulation of C-repeat binding factor/dehydration-responsive element-binding (CBF/DREB) proteins, necessary for activating cold-responsive genes, thereby inhibiting growth [80]. Cold stress hampers growth by inhibiting metabolic and physiological processes, such as water absorption, cellular dehydration, and oxidative stress [81,82]. Previous studies showed that the negative effects of cold stress during early development stages restrict leaf area expansion and reduce biomass accumulation [83,84,85]. An increased dry matter content indicates plant dehydration under cold stress. As previously documented, low temperatures can induce tissue dehydration and water deficit by reducing water absorption without decreasing leaf transpiration rates [86]. Cold acclimation involves numerous physiological and biochemical alterations, with a primary outcome being the reduction in growth [87]. Additionally, the decrease in growth could be attributed to an increase in reactive oxygen species (ROS) in plants, which negatively impacts growth [88,89].

Low temperature resulted in changes in the spectral reflectance signature in the VIS region in both crops with more pronounced differences between cold stress with and without priming in maize compared to soybean (Figure 5). In maize priming treatment, temperature mostly affected leaf spectral reflectance at 515–560 nm and 685–700 nm (Figure 5a). Cold-stressed plants had higher reflectance across these wavebands compared to plants grown in control conditions. An increase in reflectance in the VIS region indicates a decrease in pigment content [90]. While the mean reflectance signatures of maize mostly increased compared to their initial condition in control, in soybean, the reflectance slightly decreased compared to the control at a wavelength under 680 nm and increased at a longer wavelength (Figure 5b). The primary cause of these reflectance differences could be differences in pigment content, especially in chlorophyll and anthocyanin content [44]. Moreover, the ZMI, GM1, and GM2 were slightly affected by cold stress in soybean (Figure 3b,i,j), suggesting that the chlorophyll content in leaves remained stable. In leaves with consistent chlorophyll content, a reduction in leaf thickness could lead to an elevated chlorophyll concentration, thereby causing a decrease in reflectance in the visible (VIS) region in soybean [90]. In addition, changes in chlorophyll *a* fluorescence parameters under cold stress exhibited correlations ranging from weak to strong with changes in relative reflectance measured at specific wavelengths (Figure 6a–g). Moderate-to-strong correlations were observed in maize between changes in biomass traits and changes in spectral reflectance (Figure 6h,i). In contrast, changes in biomass traits showed weak correlations with changes in spectral reflectance measured at specific wavelengths in soybean (Figure 6h,i). These findings confirm a greater impact of cold stress and priming on maize.

## 4. Materials and Methods

### 4.1. Plant Material, Experimental Design, and Growth Conditions

A total of 14 dent maize inbred lines belonging to FAO 500–600 and 12 soybean cultivars from the 0–I maturity group, previously not tested for cold stress tolerance, were used in this study. All genotypes are the property of Agricultural Institute Osijek (AIO, Osijek, Croatia).

The experiment was conducted in a walk-in growth chamber, Fitoclima 10.000 HP (Aralab, Rio de Mouro, Portugal). It consisted of a control (C), cold stress (S), and cold stress after priming for two days (P) in four biological replicates for each genotype per treatment and five plants per replicate. Plants in every treatment were grown in separate time series. The cold stress initiation for S and P was planned for when each crop had a fully developed first leaf or trifoliate, i.e., 13 days after sowing (DAS) for maize and 20 DAS for soybean, which is why soybean was sown seven days ahead of maize. The time required for each crop to fully develop its first leaf or trifoliate under given conditions was previously established. The plants were grown in plastic containers (510 × 350 × 200 mm) filled with 5.5 kg of soil (pH (CaCl_3_) = 5.7, N (NH_4_^+^ + NO_3_^−^) = 70 mg/L, P (P_2_O_5_) = 50 mg/L, K (K_2_O) = 90 mg/L, EC = 40 mS/m) and divided into 12 rows with 3 and 2 planting spaces placed interchangeably for soybean and 7 rows with 5 planting spaces for maize. There were six soybean and seven maize genotypes per tray, two trays per replicate, and eight trays per treatment. The order of the genotypes per replicate was randomized, and trays were randomly shuffled in the growth chamber every day before the lights turned on. All plants were watered with tap water every other day. The growth chamber settings were tuned to 25/18 °C or 10/5 °C, depending on the treatments (Figure 1), with a 16 h/8 h (light/dark) photoperiod, 70/90% relative humidity, and 300 μmol m^−2^ s^−1^ light intensity. The growth conditions were monitored daily, and the temperature and humidity conditions of the growth chamber were recorded by the FitoLog9000 data logger (Aralab, Rio de Mouro, Portugal).

### 4.2. Chlorophyll a Fluorescence, Reflectance, and Biomass Measurements

**Figure 7:**
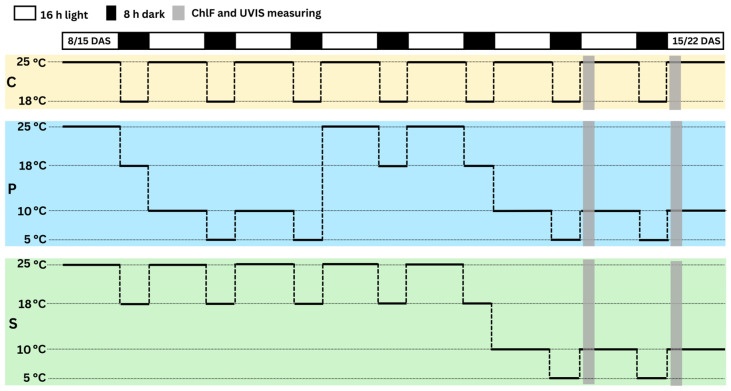
The growth chamber settings for the control (C), cold stress after two days of priming (P), and stress without priming (S) from the 8th to 15th day after sowing for maize and the 15th to 22nd day after sowing for soybean.

For all treatments, measurements were taken in the middle of the first fully developed leaf for maize and on the middle leaflet of the first fully developed trifoliate for soybean. Two measurements per genotype in each replicate were made, comprising a total of eight measurements per genotype per treatment. The measurements were taken on two consecutive days starting from the 14th DAS for maize and the 21st DAS for soybean, i.e., after 24 and 48 h of cold stress in P and S treatment (Figure 7).

The chlorophyll *a* fluorescence parameters were assessed by determining fluorescence with a saturation pulse method after 30 min dark adaptation [91] using a portable fluorometer FluorPen FP 110/D (Photon Systems Instruments, Drásov, Czech Republic). All plants were exposed to light in the growth chamber for about half an hour before dark adaptation started. The data recorded by measuring chlorophyll *a* fluorescence, expressed in relative units, were analyzed using the JIP test as previously described by Strasser et al. [55]. The parameters used for quantifying PSII behavior with equations used to calculate ChlF parameters and the explanation of parameters are shown in Table 1.

The spectral reflectance of leaves was measured using a handheld instrument PolyPen RP 410 (Photon Systems Instruments, Drásov, Czech Republic) in the UVIS response range (380–790 nm). The data recorded by measuring leaf spectral reflectance were processed with SpectraPen software (Drásov, Czech Republic) which automatically calculates several spectral reflectance indices based on the measured reflectance spectra. The spectral reflectance indices used in this research are shown in Table 2.

The aboveground biomass of all five plants per genotype per replicate was weighed on the four-decimal laboratory scale and designated as fresh mass (FM). Plants were dried for 24 h at 70 °C before weighting for dry mass. The dry matter content (DMC) was expressed as the percentage of FM.

### 4.3. Statistical Analysis

The data were analyzed using the statistical software package JASP [98]. Before statistical tests, the normality of the data was checked by using the Shapiro–Wilk test (*p* < 0.05). Throughout this manuscript, means are compared by an analysis of variance (ANOVA) followed by Tukey’s HSD post hoc test (*p* < 0.05), except for comparing two groups where a *t*-test was used. All replicates considered in our study were independent biological replicates originating from different plants. Since there was no statistically significant difference among the measured values under control conditions, the measurements from two days were pooled to simplify the data representation. The correlation between spectral reflectance wavebands and photosynthetic parameters, as well as biomass traits, was performed using the mean value of the difference between specific treatment and the control for all genotypes of each crop.

## 5. Conclusions

This study revealed crop-specific responses to cold stress and priming in maize and soybean, underscoring the importance of chlorophyll *a* fluorescence and spectral reflectance parameters in comprehending plant stress dynamics. Both crops exhibited an overall impairment of the chlorophyll a fluorescence kinetics as a result of cold stress, with distinct responses observed between maize and soybean. In maize, the negative effect of cold stress became more pronounced as the stress duration increased, whereas in soybean, it remained consistent after the initial change. Spectral reflectance indices suggested the chlorophyll content was less affected by cold stress in soybeans than in maize. However, photoprotective pigments’ increase in both crops indicated plant protection mechanisms were initiated. The spectral reflectance signature under cold stress varied not only among treatments but among genotypes as well, indicating the possibility of cold stress response variability necessary for plant breeding. The difference between cold stress with and without priming was more evident in maize. Although for most fluorescence parameters and spectral reflectance indices in maize priming resulted in a cumulatively negative effect, the electron trapping efficiency was stabilized and the electron transfer system upregulated, indicating an adaptive response protecting the photosystems from more significant stress-induced damage. A correlation analysis showed weak-to-moderate correlations between chlorophyll *a* fluorescence parameters, reflectance, and biomass traits in both crops, confirming a greater impact of cold stress and priming on maize than soybean.

The present study is the first one involving crops with diverse photosynthetic pathways to understand the effects of cold stress in early development under controlled conditions. Understanding the complex physiological effects of unfavorable external events in plant production is the first step in mitigating them. Further research is needed to determine the genotype-specific cold stress response and the effect of priming in initiating stress memory in maize and soybeans, aiming to facilitate the decision-making in breeding programs directed towards increasing cold stress tolerance.

## Figures and Tables

**Table 1:** Chlorophyll *a* fluorescence parameters, equations, and parameter definitions according to Strasser et al. [55] and Yusuf et al. [56]. F_v_ is maximum variable fluorescence, F_m_ is maximum fluorescence intensity, V_J_ is relative variable fluorescence at J-step, V_I_ is relative variable fluorescence at I-step, M_0_ is initial slope of O-J fluorescence rise, PSI is photosystem I, PSII is photosystem II, and QA is first plastoquinone electron acceptor of PSII.

Parameter Equation	Definition
Efficiencies and quantum yields:	
${TR}_{0}/ABS=F_{v}/F_{m}$	Maximum quantum yield of primary PSII photochemistry
${ET}_{0}/{TR}_{0}=1-V_{J}$	Efficiency with which PSII trapped electron is transferred from Q_A_^−^ to PQ
${RE}_{0}/{TR}_{0}=1-V_{I}$	Efficiency with which PSII trapped electron is transferred to final PSI acceptors
${RE}_{0}/{ET}_{0}=\left({RE}_{0}/{TR}_{0}\right)/\left({ET}_{0}/{TR}_{0}\right)$	Efficiency with which electron from PQH_2_ is transferred to final PSI acceptors
${ET}_{0}/ABS=\left({TR}_{0}/ABS\right)\times \left({ET}_{0}/{TR}_{0}\right)$	Quantum yield of electron transport from Q_A_^−^ to PQ
${RE}_{0}/ABS=\left({TR}_{0}/ABS\right)\times \left({RE}_{0}/{TR}_{0}\right)$	Quantum yield of electron transport from Q_A_^−^ to final PSI acceptors
Performance indices:	
${PI}_{abs}=RC/ABS\times \frac{{TR}_{0}/ABS}{1-{TR}_{0}/ABS}\times \frac{{ET}_{0}/{TR}_{0}}{1-{ET}_{0}/{TR}_{0}}$	Performance index on absorption basis
${PI}_{total}={PI}_{abs}\times \frac{{RE}_{0}/{ET}_{0}}{1-{RE}_{0}/{ET}_{0}}$	Total performance index on absorption basis
Specific energy fluxes:	
$ABS/RC=\left(M_{0}/V_{j}\right)\times \left[1/\left({TR}_{0}/ABS\right)\right]$	Apparent antenna size of active PSII
${TR}_{0}/RC=M_{0}/V_{J}$	Maximum trapped exciton flux per active PSII
${ET}_{0}/RC=\left(M_{0}/V_{J}\right)\times \left({ET}_{0}/{TR}_{0}\right)$	Flux of electrons transferred from Q_A_^−^ to PQ per active PSII
${RE}_{0}/RC=\left(M_{0}/V_{J}\right)\times \left({RE}_{0}/{TR}_{0}\right)$	Flux of electrons transferred from Q_A_^−^ to final PSI acceptors per active PSII

**Table 2:** Chosen spectral reflectance indices.

Abbreviation	Index Name	Equation	Reference
PRI	Photochemical Reflectance Index	(R_531_ − R_570_)/(R_531_ + R_570_)	[92]
ZMI	Zarco-Tejada and Miller Index	R_750_/R_710_	[93]
CRI1	Carotenoid Reflectance Index	(1/R_510_) − (1/R_550_)	[94]
CRI2		(1/R_510_) − (1/R_700_)	
ARI1	Anthocyanin Reflectance Index	(1/R_550_) − (1/R_700_)	[78]
ARI2		R_800_x[(1/R_550_) − (1/R_700_)]	
Ctr1	Carter Index	R_695_/R_420_	[95]
Ctr2		R_695_/R_760_	[96]
GM1	Gitelson and Merzlyak Index	R_750_/R_550_	[97]
GM2		R_750_/R_700_	

## Data Availability

The raw data supporting the conclusions of this article will be made available by the authors on request.
